# Effect of power arm length and attachment configuration on maxillary molar mesialization with clear aligners: a finite element analysis

**DOI:** 10.1186/s12903-026-09893-0

**Published:** 2026-09-22

**Authors:** Ahmet Cavusoglu, Buket Erdem

**Affiliations:** Department of Orthodontics, Faculty of Dentistry, Istanbul Health and Technology University, Istanbul, Turkey

**Keywords:** Finite element analysis, Clear aligners, Molar mesialization, Power arm

## Abstract

**Background:**

Maxillary molar mesialization with clear aligners is challenging because achieving controlled root movement without excessive mesial tipping remains difficult. Although power arms can modify the force system, the combined effects of power-arm length and attachment configuration on molar displacement and tipping remain insufficiently characterized. This study evaluated the effects of 6-, 8-, and 10-mm power arms combined with two attachment configurations during maxillary first molar mesialization using three-dimensional finite element analysis.

**Methods:**

The attachment configurations were selected to represent two clinically distinct approaches to integrating a vertical attachment with a power arm: a palatal vertical attachment combined with a buccal cut-out and a buccal vertical attachment with direct power-arm integration. Six three-dimensional finite element models were constructed by combining three power arm lengths (6, 8, and 10 mm) with two attachment configurations: a palatal vertical attachment with a buccal cut-out, and a buccal vertical attachment without a cut-out. A mesial force of 200 g was applied from a temporary anchorage device (TAD) to the power arm. Displacements were measured along the X (bucco–palatal), Y (mesio–distal), and Z (occluso–gingival) axes. Peak von Mises stress values in the periodontal ligament and aligner deformation were evaluated.

**Results:**

In all models, maxillary first molar displacement occurred predominantly in the mesial and gingival directions. Mesial crown displacement ranged from 0.068 to 0.074 mm, while distal displacement at the root apex ranged from 0.011 to 0.012 mm. Mesial tipping angles ranged from 0.155° to 0.170°, with higher tipping observed in models with shorter power arms. Increasing power arm length was consistently associated with reduced mesial tipping and lower periodontal ligament stress, but with reduced mesial crown displacement. Shorter power arms generated higher periodontal ligament stress, whereas longer power arms were associated with greater aligner deformation.

**Conclusions:**

Power arm length and attachment configuration were associated with differences in initial tooth displacement patterns, periodontal ligament stress distribution, and aligner deformation during maxillary first molar mesialization with clear aligners. Longer power arms were associated with reduced mesial tipping and lower periodontal ligament stress, but also with reduced mesial crown displacement. The buccal vertical attachment configuration consistently produced lower mesial rotation than the palatal vertical attachment with buccal cut-out configuration at each corresponding power-arm length; this difference should be interpreted as a configuration-dependent finding within the initial biomechanical response.

## Background

Clear aligner therapy has become an established treatment modality for a broad range of orthodontic tooth movements [1–5]. However, achieving controlled maxillary molar mesialization remains challenging because the applied force system may produce mesial crown tipping rather than bodily tooth movement, thereby compromising treatment predictability [6, 7]. Effective mesialization therefore requires appropriate control of the relationship between the force vector and the molar’s center of resistance (CR) to limit unwanted tipping and maintain root control [6].

Current compensatory strategies, such as specialized attachments and staging protocols, have shown variable clinical success, and high-level evidence supporting their predictability remains limited [8–11]. Recent clinical evidence has further confirmed these limitations. Lin et al. [7] reported that posterior tooth mesialization with clear aligners in moderate anchorage cases showed limited predictability, with significant molar mesial tipping and discrepancies between planned and achieved movements, particularly in the maxillary arch.

In fixed appliance therapy, power arms modify the vertical position of force application and consequently alter the line of action of the applied force relative to the tooth’s CR [8]. This change modifies the resulting moment-to-force relationship and can therefore influence the balance between crown tipping and bodily tooth displacement during mesialization. Accordingly, the biomechanical effect of power-arm length may depend on the resulting force application geometry and its relationship to the molar’s CR. Although power-arm support has been predominantly investigated for distalization movements in conjunction with clear aligner therapy [9–11], recent finite element studies have examined different biomechanical approaches to molar mesialization with clear aligners, including TAD-supported auxiliary mechanics, attachment design, and tipping compensation, providing insights into molar displacement patterns, root engagement, and tipping control [12–14]. However, the effects of different power-arm lengths in combination with different attachment configurations during TAD-supported maxillary first molar mesialization remain insufficiently characterized.

None of the available studies have directly compared multiple power-arm lengths across different attachment configurations within a single systematic model. Because power-arm length can modify the vertical position of the force vector and alter the moment-to-force relationship at the molar center of resistance, whereas attachment configuration may influence aligner–tooth engagement and force transmission geometry, understanding their combined effects is biomechanically relevant for controlled molar mesialization.

Finite element analysis (FEA) is a computational method that enables the simulation of stress distribution, periodontal ligament (PDL) response, and initial tooth displacement under defined loading conditions [15]. FEA provides a simulated initial biomechanical response to applied forces and does not directly represent long-term clinical tooth movement or biological adaptation [16].

Therefore, the present study evaluated the initial biomechanical effects of 6-, 8-, and 10-mm power arms combined with two attachment configurations during maxillary first molar mesialization using three-dimensional finite element analysis, focusing on tooth displacement, tipping and rotation, periodontal ligament stress, and aligner deformation.

## Methods

### Study design and computational environment

The study was designed and supervised at the Department of Orthodontics, Faculty of Dentistry, Istanbul Health and Technology University, Istanbul, Turkey. The authors defined the study design, model configurations, loading protocol, and boundary conditions, and interpreted all results. Three-dimensional geometric reconstruction, solid model generation, finite element model development, and stress analyses were performed by Tinus Engineering (Ankara, Turkey) on a workstation equipped with an 11th Generation Intel^®^ Core™ i9-11900 processor (2.50 GHz) and 64 GB ECC RAM.

### Acquisition of anatomical data and maxillary bone modeling

The maxillary bone model was developed based on computed tomography (CT) data obtained from the Visible Human Project (National Library of Medicine, Bethesda, MD, USA). The CT dataset was reconstructed with a slice thickness of 0.33 mm and exported in DICOM format. Image segmentation was performed using 3D Slicer software (version 5.6.2; Brigham and Women’s Hospital, Boston, MA, USA), where the cortical and trabecular bone structures were isolated using predefined Hounsfield unit thresholds (426.50–3193.04). Unwanted regions and imaging artifacts were manually removed to ensure anatomical accuracy.

Following segmentation, the anatomical structures were converted into three-dimensional surface models and exported in STL format. The trabecular bone model was generated by referencing the internal surface of the cortical bone, while the mucosal layer was created by applying a uniform outward offset to the cortical bone surface. The mucosal layer was not included in the finite element model because the present analysis focused on the mechanical response of the teeth, periodontal ligament, alveolar bone, aligner, and orthodontic components under the applied loading conditions. The PDL was modeled by applying a uniform 0.25 mm offset from the external root surfaces of the teeth [17]. All anatomical components were positioned within a unified three-dimensional coordinate system using Blender software (Blender Foundation, Amsterdam, Netherlands).

### Modeling of clear aligners, attachments and power arms

The clear aligners, attachments, power arms, and buttons were digitally designed using Blender software based on predefined geometric dimensions. Each power arm consisted of a vertical shaft with a rectangular cross-section and a rounded loop at its apical end, which served as the point of elastic force application. In the button-supported configuration, the button had a diameter of 3 mm. The three power-arm configurations differed in length (6, 8, and 10 mm), while the remaining geometric characteristics were kept constant. For all simulation models, the clear aligner was modeled with a uniform thickness of 0.75 mm by offsetting the external surfaces of the teeth and attachments.

**Fig. 1:**
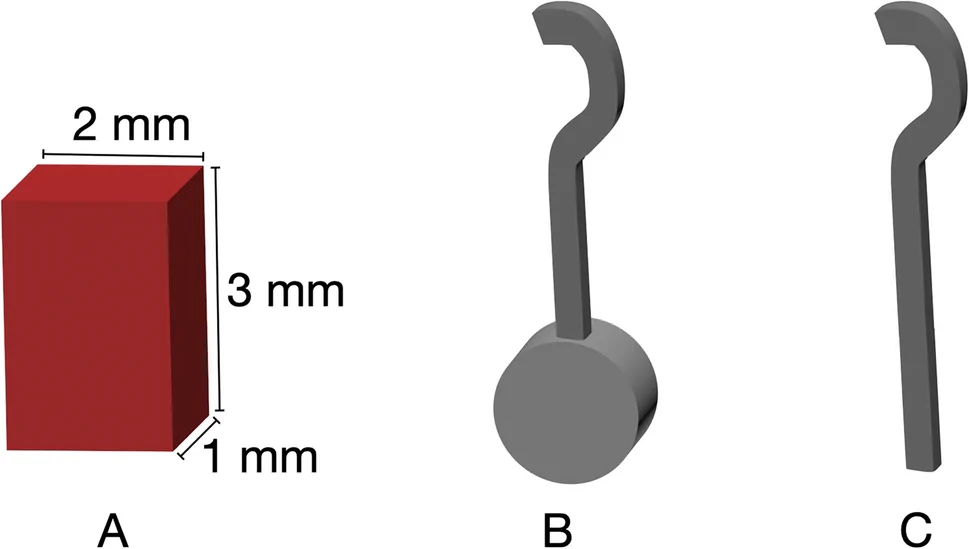
Geometric design of the rectangular vertical attachment (3 mm height × 2 mm width × 1 mm thickness) and of the three power arms (6, 8, and 10 mm) modelled with and without buttons. **A** rectangular vertical attachment, **B** power arm with a button, and **C** power arm without a button

Rectangular vertical attachments were modeled with dimensions of 3 mm in height, 2 mm in width, and 1 mm in thickness (Fig. 1). Power arms were created with and without buttons (Fig. 1). The power arm and vertical attachment were modeled as separate components, with a freeze contact defined at their interface to prevent relative motion, and were assigned distinct material properties. The vertical attachment was modeled with an elastic modulus of 12,500 MPa and a Poisson’s ratio of 0.36, whereas the power arm was modeled with an elastic modulus of 193,000 MPa and a Poisson’s ratio of 0.30 (Table 1). In Models 1–3, a rounded, elongated buccal cut-out was incorporated into the aligner to provide clearance for the power arm and its button. The dimensions of the cut-out were determined according to the dimensions of the button and were kept constant across the 6-, 8-, and 10-mm power-arm models. In Models 4–6, no buccal cut-out was incorporated because the power arm was integrated with the buccal vertical attachment.

**Table 1:** Material properties of the components

Material	Elastic Modulus (MPa)	Poisson Ratio (v)
Cortical bone	13,700	0.30
Cancellous bone	1,370	0.30
Aligner	528	0.36
Attachment	12,500	0.36
Power arm	193,000	0.30
Teeth	19,600	0.30
PDL	0.67	0.45

### Finite element model configurations

Six distinct finite element models were generated by varying the power arm length and attachment configuration. In all models, a mesial force of 200 g was applied from a TAD toward the power arm, simulating maxillary first molar mesialization. Each aligner was programmed to produce a 0.2 mm mesial displacement per stage toward the second premolar extraction space.

Two attachment configurations were modeled: a palatal vertical attachment combined with a buccal cut-out and a buccal vertical attachment with direct power-arm integration. These configurations were modeled as predefined, distinct configurations, and no independent factorial analysis of attachment location, buccal cut-out presence, or power-arm integration was performed.

**Fig. 2:**
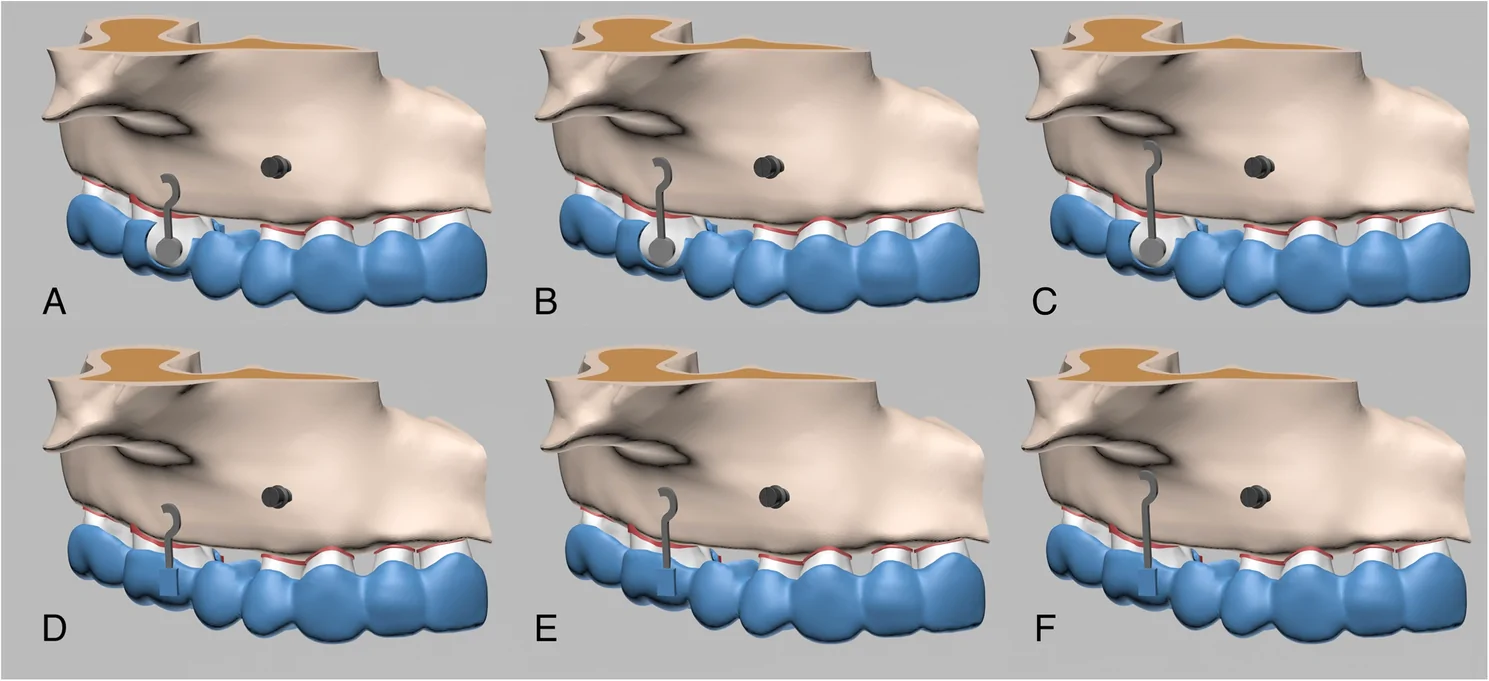
Overview of the six finite element models generated by combining three power arm lengths (6, 8, and 10 mm) with two attachment configurations (palatal vertical attachment with buccal cut-out; buccal vertical attachment without cut-out)

All finite element models evaluated in this study are illustrated in Fig. 2.

**Fig. 3:**
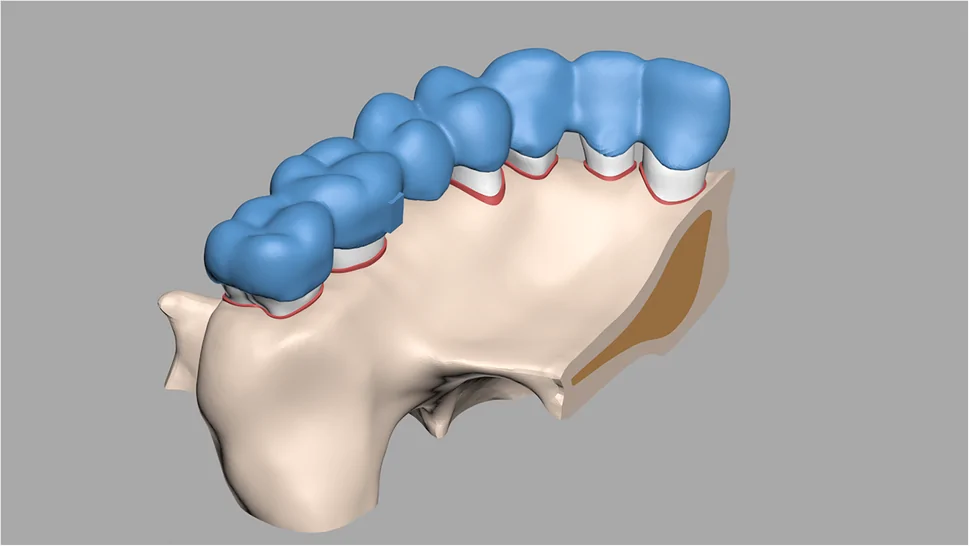
Representative model (Model 1) illustrating the 6 mm power arm positioned within the buccal cut-out region of the aligner together with the palatal vertical attachment

- Model 1 (A): 6 mm power arm located in a buccal cut-out region with a palatal vertical attachment (Fig. 3)
- Model 2 (B): 8 mm power arm located in a buccal cut-out region with a palatal vertical attachment
- Model 3 (C): 10 mm power arm located in a buccal cut-out region with a palatal vertical attachment
- Model 4 (D): 6 mm power arm integrated with a buccal vertical attachment
- Model 5 (E): 8 mm power arm integrated with a buccal vertical attachment
- Model 6 (F): 10 mm power arm integrated with a buccal vertical attachment

### Mesh generation and mathematical model development

**Fig. 4:**
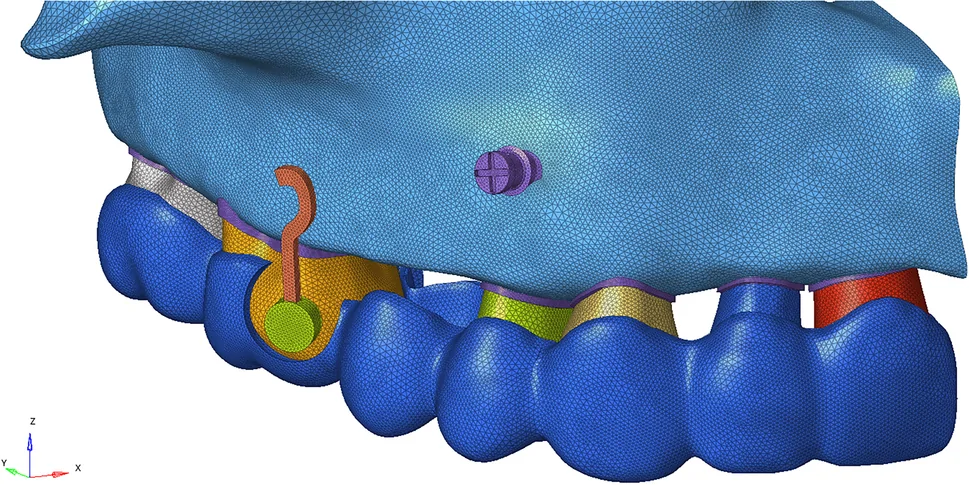
Finite element mesh of the model components

After geometric modeling, all components were imported into Altair HyperMesh software (Altair, Troy, MI, USA) for finite element discretization. Surface meshes were generated using triangular elements with sizes ranging from 0.1 mm to 0.25 mm (Fig. 4). Volumetric meshes were created using tetrahedral solid elements to ensure accurate stress and displacement calculations. Mesh density was determined through a formal mesh-convergence analysis. Models with element sizes of 0.40, 0.30, 0.20, 0.15, and 0.10 mm were analyzed under identical loading and boundary conditions, using maximum displacement magnitude as the convergence parameter. The relative error between successive mesh refinements decreased to 2.51% at 0.15 mm and 0.62% at 0.10 mm. Because the difference between successive solutions was below 3% at 0.15 mm and smaller element sizes, mesh independence was considered to have been achieved. The resulting finite element models were transferred to Altair OptiStruct (Altair, Troy, MI, USA) for numerical analysis.

### Material properties

Cortical and trabecular bones were modeled as isotropic materials, and the teeth, periodontal ligament, clear aligners, attachments, and power arms were assumed to be homogeneous and isotropic. All materials were defined as linearly elastic. The elastic modulus and Poisson’s ratio values used in the simulations are presented in Table 1.

### Loading conditions and boundary constraints

**Fig. 5:**
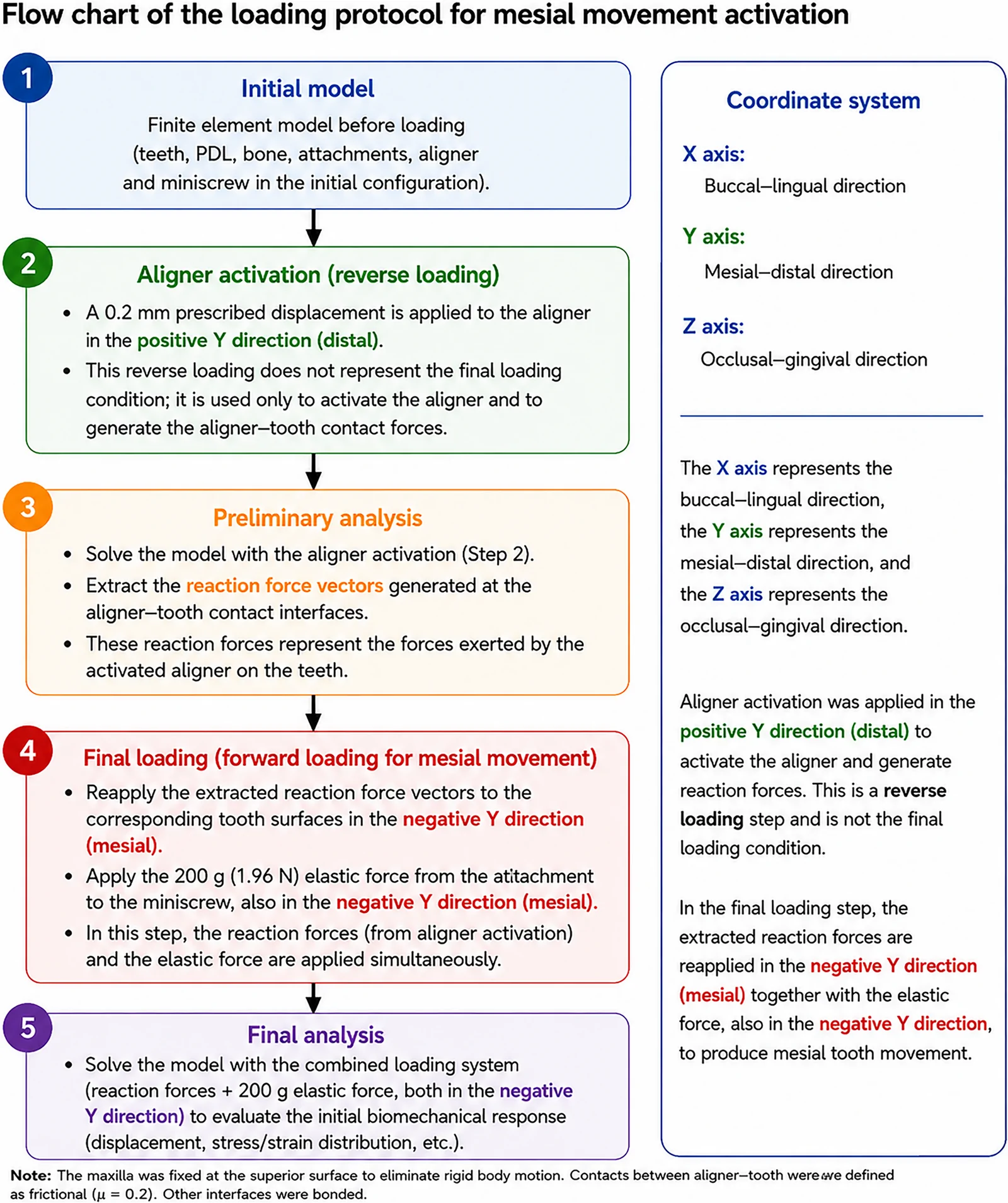
Schematic representation of the two-step loading protocol

The mesial movement was activated using the same loading strategy described by Gao et al. [18], with modifications according to the present study (Fig. 5). First, a 0.2 mm prescribed distal displacement was applied to the aligner to simulate the programmed tooth movement. This prescribed displacement generated the aligner deformation required for force delivery. Subsequently, in the elastic-assisted models, a 200 g (1.96 N) mesially directed elastic force was applied from the attachment toward the TAD.

A two-step finite element procedure was employed, as illustrated in Fig. 5. Following preliminary aligner activation, the reaction forces generated at the aligner–tooth interfaces were extracted and subsequently reapplied to the corresponding contact surfaces in the opposite direction to reproduce the force system generated by the programmed aligner activation. The final finite element analysis was then performed under the combined loading condition.

**Fig. 6:**
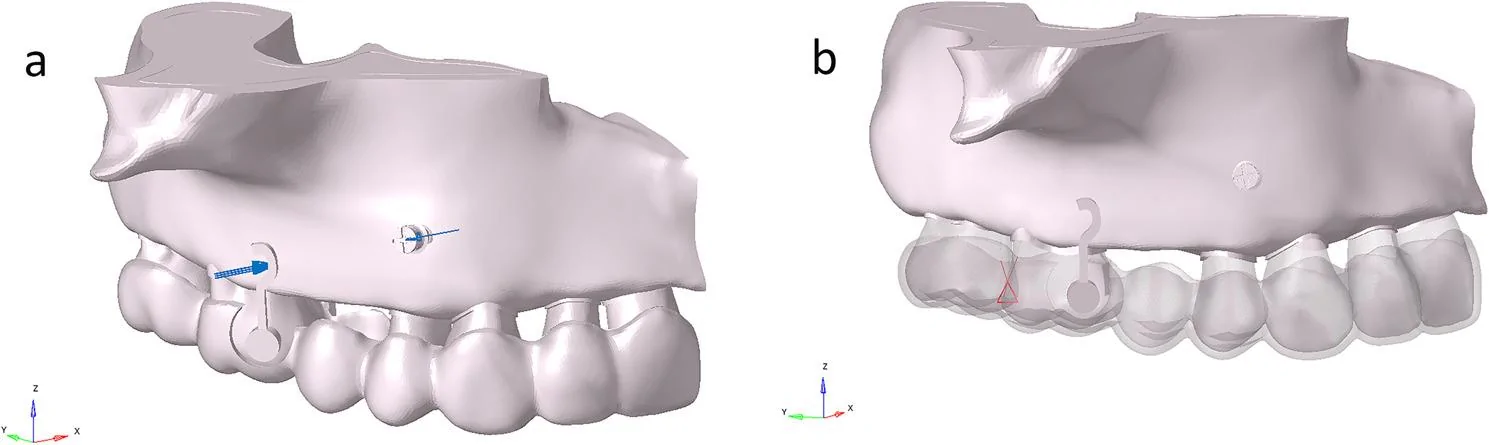
Position of the temporary anchorage device (TAD) between the canine and the premolar, defining the point and line of action of the 200 g mesially directed elastic force applied to the power arm. **a** oblique view showing the TAD position and force direction; **b** lateral view showing the relationship between the TAD and the power arm

The TAD was included solely to define the clinical point and line of action of the elastic force and was not evaluated as a structure of interest. The elastic force was applied between the hook at the end of the power arm and the TAD, reproducing the corresponding force vector and moment generated by the elastic (Fig. 6). Accordingly, no stress or displacement analyses were performed for the TAD or the bone–implant interface.

Boundary conditions were defined by constraining all degrees of freedom at the superior region of the maxilla to prevent rigid-body motion; this fixation did not restrict the physiological displacement of the teeth within the periodontal ligament. Symmetric boundary conditions were applied in the Y–Z plane, while normal force components were assigned along the X-axis.

For all finite element models, the X axis represents the bucco–palatal direction, the Y axis represents the mesio–distal direction, and the Z axis represents the occluso–gingival direction. The prescribed aligner displacement and the elastic force were both applied primarily along the negative Y axis to produce mesial tooth movement. Accordingly, the reported displacement components in the X, Y, and Z directions correspond to the decomposition of the three-dimensional tooth movement resulting from the applied loading conditions, rather than indicating conflicting boundary constraints.

### Contact definitions

Frictional contact with a coefficient of friction of 0.2 was defined at the aligner–tooth and aligner–attachment interfaces. Freeze contact was defined at the tooth–PDL–bone interfaces and between the remaining connected orthodontic components, preventing relative motion between the corresponding surfaces.

### Quantitative model characteristics

Each finite element model consisted of approximately 750,000 nodes and 3.55 million elements. The detailed node and element counts for all six models are summarized in Table 2. A total of six nonlinear static analyses were performed under the defined loading and boundary conditions.

**Table 2:** Number of nodes and elements

	Total number of nodes	Total number of elements
Model 1	750,046	3,550,408
Model 2	750,192	3,550,912
Model 3	750,487	3,552,229
Model 4	750,214	3,551,007
Model 5	750,360	3,551,511
Model 6	750,655	3,552,828

### Statistical analysis

Because the finite element simulations were deterministic numerical analyses of predefined model configurations rather than observations from a sampled population, inferential statistical testing was not performed. Differences between configurations were evaluated directly from the numerical outputs of the simulations. Model verification, including assessment of mesh adequacy and numerical convergence, was considered separately from statistical inference.

### Outcome measurements and calculations

Translational displacement was evaluated at two predefined anatomical landmarks of the maxillary first molar: the mesiobuccal cusp tip as the crown reference point and the mesiobuccal root apex as the root reference point, consistent with the landmark approach used in previous finite element studies of molar displacement with clear aligners [19]. Displacement was resolved along the predefined three-dimensional coordinate system, with the X-axis representing the bucco–palatal direction, the Y-axis representing the mesio–distal direction, and the Z-axis representing the occluso–gingival direction. The reported crown and root displacement values refer specifically to these landmarks and their respective directional displacement components. The mesial tipping angle was determined from the differential mesio-distal displacement between the crown and root reference points, using the 18-mm distance between these landmarks as the reference tooth length. The tipping angle was calculated as θ = arctan[(Δyᶜ − Δy_r_)/18], where Δyᶜ and Δy_r_ represent the mesio-distal displacements of the mesiobuccal cusp tip and mesiobuccal root apex, respectively. Rotational displacement was evaluated using a rectangular reference defined by the molar cusp landmarks, with the short side (h = 7.2 mm) defined as the distance between the mesiobuccal and mesiopalatal cusps and the long side (l = 10.5 mm) as the distance between the mesiobuccal and distobuccal cusps. The rotational angle (θ) was calculated as θ = arctan(Δx/h), where Δx represents the difference in bucco-palatal displacement between the mean displacements of the upper and lower edges of the reference rectangle.

## Results

Displacements of the maxillary first molar were quantified as total displacement and as directional components along the X (bucco–palatal), Y (mesio–distal), and Z (occluso–gingival) axes. Measurements were obtained at the mesiobuccal cusp tip and mesiobuccal root apex landmarks, together with peak von Mises stress values in the PDL and aligner deformation.

**Fig. 7:**
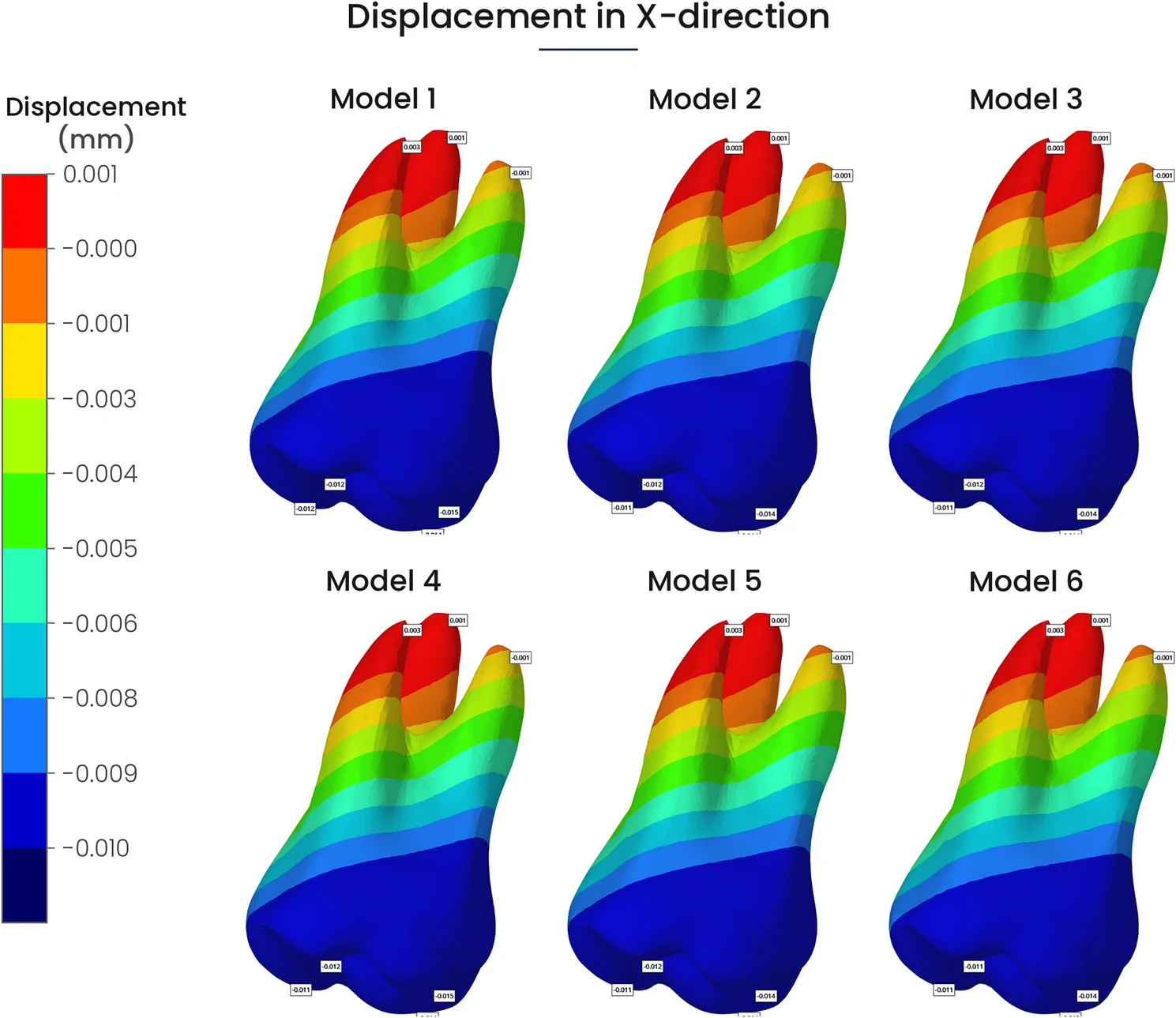
Displacement of the maxillary first molar along the X axis (bucco–palatal direction) in the six finite element models. The colour scale bar indicates displacement magnitude in millimetres

**Fig. 8:**
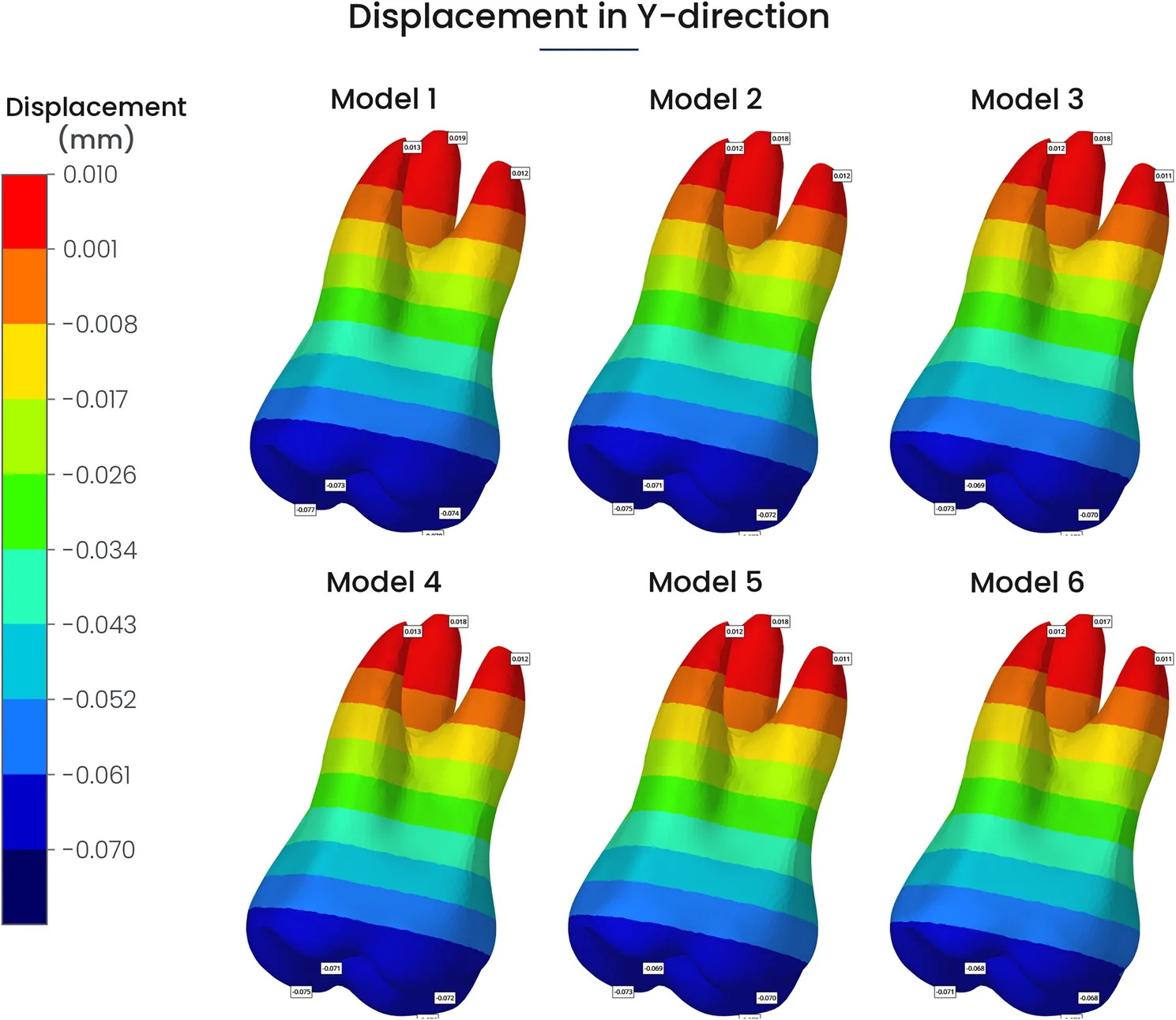
Displacement of the maxillary first molar along the Y axis (mesio–distal direction) in the six finite element models. The colour scale bar indicates displacement magnitude in millimetres

**Fig. 9:**
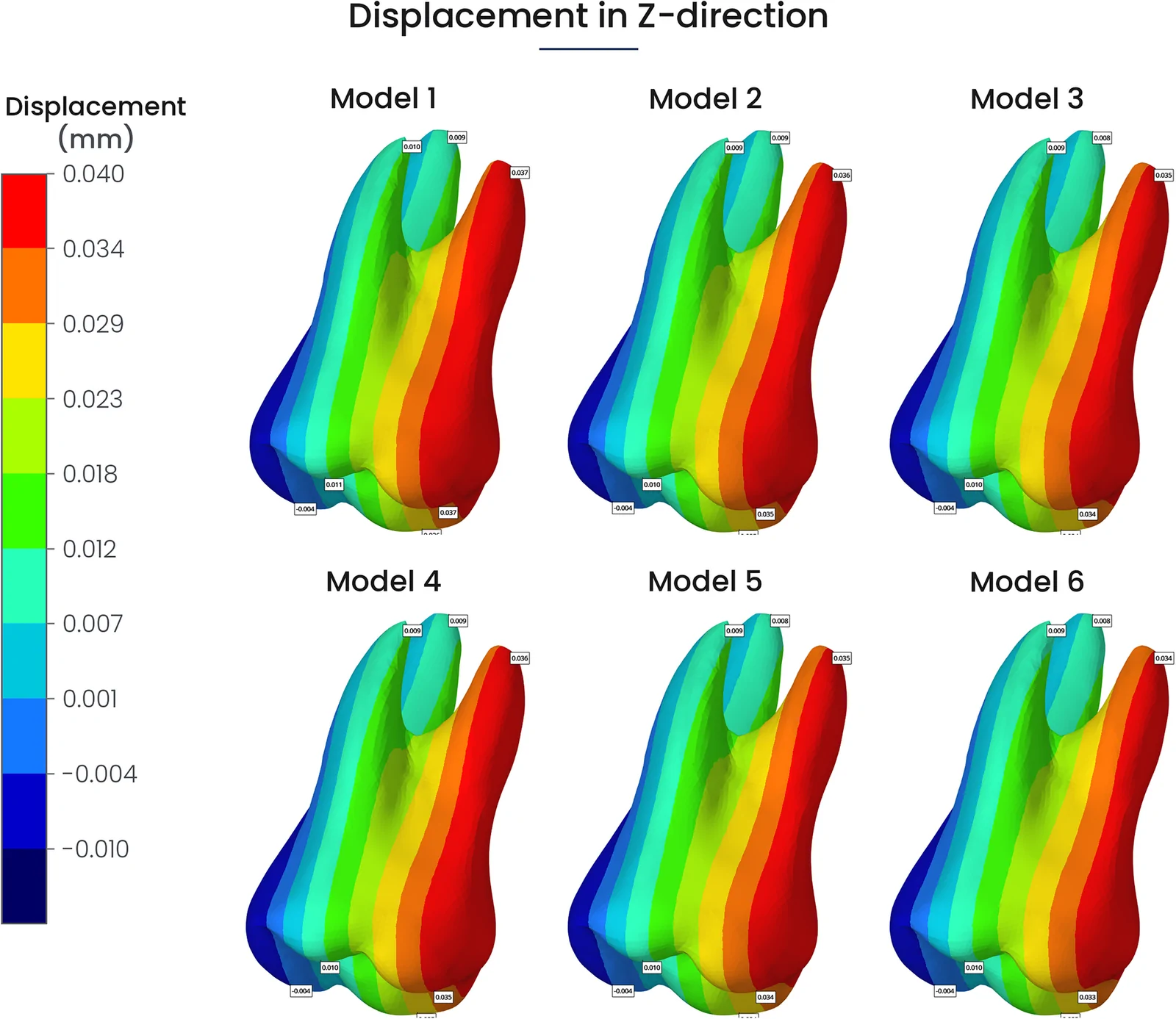
Displacement of the maxillary first molar along the Z axis (occluso–gingival direction) in the six finite element models. The colour scale bar indicates displacement magnitude in millimetres

The displacement patterns of the maxillary first molar along the X, Y, and Z axes are shown in Figs. 7, 8 and 9, and the corresponding numerical values are summarized in Table 3.

**Table 3:** Directional displacement values (mm), tipping (degree) and rotation (degree) of the maxillary first molar at crown and root apex landmarks for each finite element model

Direction	Location	Model 1	Model 2	Model 3	Model 4	Model 5	Model 6
X	Crown	-0.015	-0.014	-0.014	-0.015	-0.014	-0.014
	Apex	-0.001	-0.001	-0.001	-0.001	-0.001	-0.001
Y	Crown	-0.074	-0.072	-0.070	-0.072	-0.070	-0.068
	Apex	0.012	0.012	0.011	0.012	0.011	0.011
Z	Crown	0.037	0.035	0.034	0.035	0.034	0.034
	Apex	0.037	0.036	0.035	0.036	0.035	0.033
Mesial tipping (°)		0.170	0.164	0.161	0.164	0.161	0.155
Mesial rotation (°)		0.011	0.016	0.016	0.006	0.008	0.008

Negative and positive values indicate displacement direction according to the predefined coordinate system: negative X denotes buccal and positive X palatal displacement; negative Y denotes mesial and positive Y distal displacement; and positive Z denotes gingival displacement

The maxillary first molar exhibited mesial crown displacement in all six models, with the mesial component ranging from 0.068 to 0.074 mm. In contrast, the mesiobuccal root apex exhibited distal displacement in all models, ranging from 0.011 to 0.012 mm. Gingival displacement at the crown ranged from 0.034 to 0.037 mm, whereas mesial displacement was approximately twice as large. This crown–root displacement pattern was consistent with residual mesial tipping rather than bodily mesialization. Mesial tipping decreased progressively from 0.170° in Model 1 to 0.161° in Model 3 and from 0.164° in Model 4 to 0.155° in Model 6. Mesial rotation was 0.011°, 0.016°, and 0.016° in Models 1–3 and 0.006°, 0.008°, and 0.008° in Models 4–6, respectively.

**Fig. 10:**
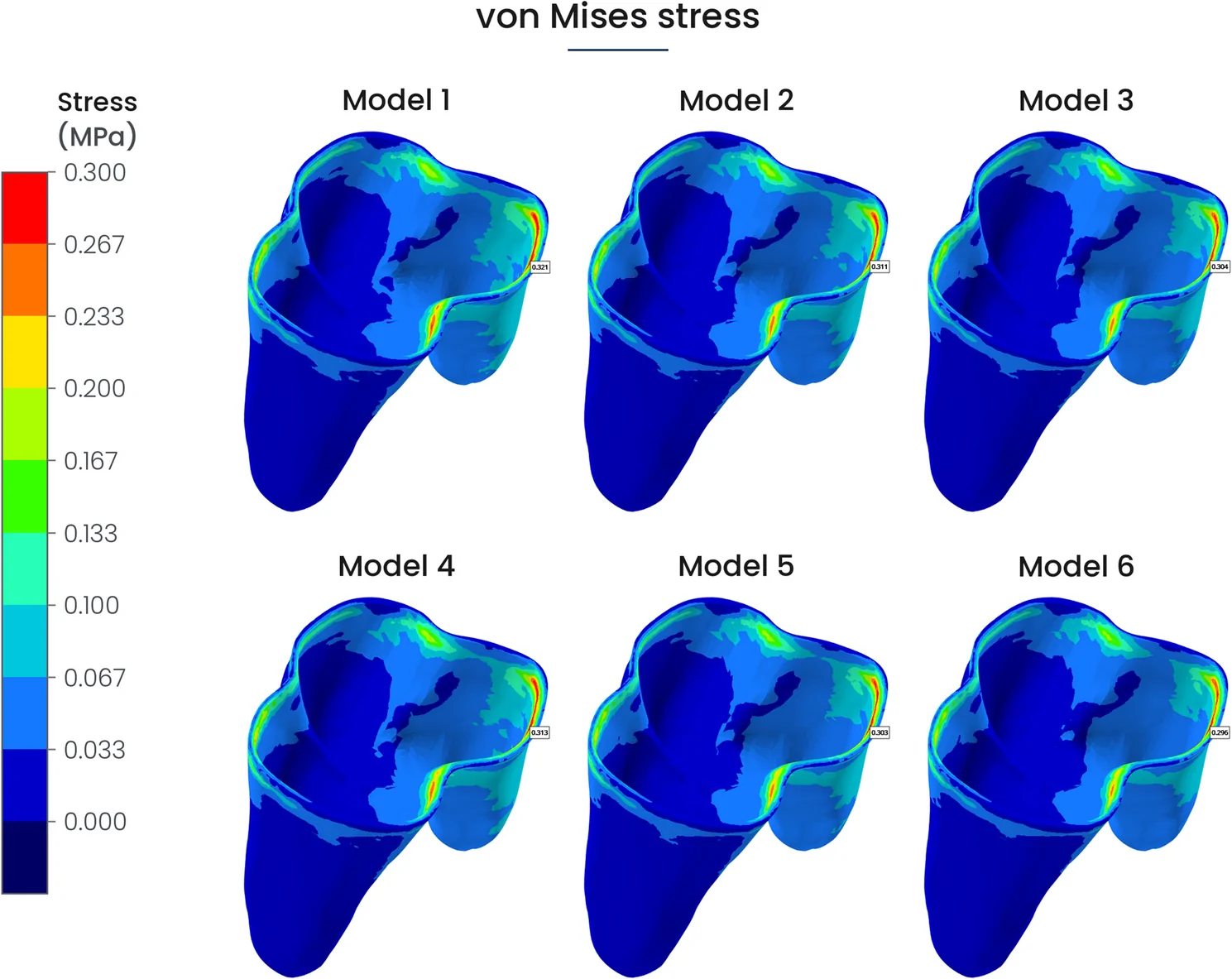
Peak von Mises stress distribution in the periodontal ligament of the maxillary first molar in the six finite element models. The colour scale bar indicates stress magnitude in MPa

Peak von Mises stress values in the PDL are reported in Table 4. Stress values ranged from 0.296 to 0.321 MPa, with the lowest stress observed in Model 6 and the highest stress in Model 1 (Fig. 10).

**Table 4:** Peak von Mises stress values in the PDL

Model	Peak von Mises stress (MPa)
Model 1	0.321
Model 2	0.311
Model 3	0.304
Model 4	0.313
Model 5	0.303
Model 6	0.296

**Fig. 11:**
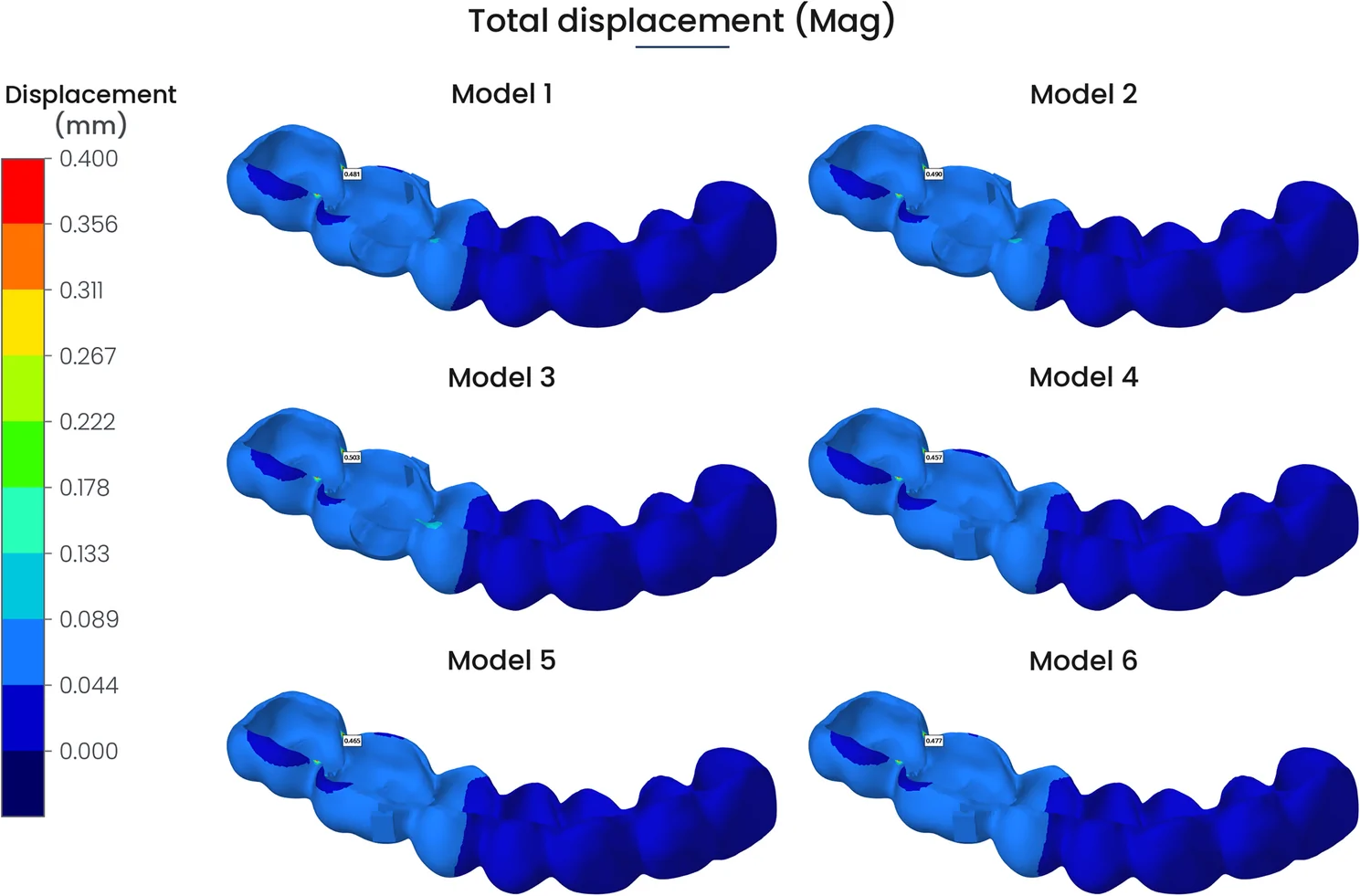
Deformation of the clear aligner in the six finite element models. The colour scale bar indicates deformation magnitude in millimetres

Aligner deformation values are presented in Table 5. Model 1 demonstrated the lowest deformation, whereas Model 3 exhibited the highest deformation (Fig. 11).

**Table 5:** Aligner deformation values

Model	Aligner deformation (mm)
Model 1	0.311
Model 2	0.490
Model 3	0.503
Model 4	0.457
Model 5	0.465
Model 6	0.477

## Discussion

The principal finding of this study was that increasing power-arm length from 6 to 10 mm was associated with lower mesial tipping and lower peak PDL stress, but also with reduced mesial crown displacement. In all models, the crown displaced mesially while the mesiobuccal root apex displaced distally, indicating that the initial biomechanical response remained characterized by mesial tipping. The two attachment configurations also showed different rotational responses: the buccal vertical attachment configuration without a buccal cut-out produced lower mesial rotation than the palatal vertical attachment configuration with a buccal cut-out at corresponding power-arm lengths. However, because attachment location, cut-out presence, and power-arm integration differed simultaneously between these configurations, this difference should be interpreted as a configuration-dependent biomechanical response rather than as an independent effect of any single component.

Among recent finite element studies, Wang et al. [12] and Lyu et al. [20] demonstrated that modifications in the auxiliary force-delivery system can substantially influence the displacement pattern during molar mesialization with clear aligners. Wang et al. [12], using a maxillary first molar model, reported that a TAD-supported power-arm system produced a more favorable displacement pattern and a relatively uniform hydrostatic PDL stress distribution compared with buccal and aligner-based buttons. Lyu et al. [20], using a mandibular molar model, showed that a modified lever arm reduced mesial tipping and rotation and promoted mesial root displacement compared with conventional button and clear aligner mechanics alone. Although these studies evaluated different auxiliary designs rather than systematically varying power-arm length, they highlight the influence of force-delivery geometry on the initial biomechanical response during molar mesialization.

Building on these observations, the present study further examined whether modifying power-arm length within a TAD-supported force-delivery system would alter the initial biomechanical response. The shortest power-arm configuration demonstrated greater mesial crown displacement but was associated with increased mesial tipping. As power-arm length increased, mesial tipping and periodontal ligament stress decreased progressively, whereas mesial crown displacement was reduced. Although the biomechanical environment of clear aligner therapy differs from that of fixed appliances, comparable directional trends in molar tipping with increasing extension-arm length have been reported in fixed appliance mechanics. Nihara et al. [21], in a finite element study of mandibular molar protraction using miniscrews and extension arms with lengths ranging from 2 to 10 mm, reported that increasing arm length progressively reduced mesial tipping, although the longest arm tested occasionally resulted in slight counter-directional tipping. Although their model used fixed appliances in a mandibular context rather than clear aligners for maxillary molar mesialization, the directional relationship between arm length and tipping tendency is consistent with the present findings.

In addition to the mesiodistal displacement pattern, a consistent buccal crown displacement component was observed across all six models during molar mesialization. Because the force was applied buccally via the power arm and TAD-supported elastic, the resultant force vector passed buccal to the molar center of resistance, contributing to a buccal displacement tendency. Wang et al. [12] similarly reported buccal displacement tendencies in their power-arm model during maxillary first molar mesialization, and comparable buccal displacement components have been reported in finite element studies of TAD-supported canine retraction using buccally positioned power arms [9]. Buccal tipping was more pronounced in models with shorter power arms, whereas increasing power-arm length was associated with a reduction in buccal displacement. This pattern may be related to the placement of the power arm. Because the power arm was positioned at an angle to prevent the hook from contacting the gingival tissues, increasing its length altered the spatial position of the force application point relative to the molar center of rotation. This geometric change may have increased the moment arm of the applied force and thereby contributed to the reduced buccal tipping tendency observed with longer power-arm configurations. Clinically, this relationship may be particularly relevant in individuals with thicker buccal soft tissues or greater alveolar bone thickness, where a greater angulation of the power arm may be required to avoid soft-tissue impingement. Such anatomical variations could further alter the spatial position of the force application point and should therefore be considered when evaluating the potential for unwanted buccal displacement.

Attachment configuration also influenced the initial biomechanical response, particularly rotational behavior. The difference in rotational response between the two attachment configurations may be related to their different spatial arrangements within the same attachment type. The power arm was directly integrated into the buccal vertical attachment in the configuration without a cut-out, whereas the buccal cut-out configuration required the same vertical attachment to be positioned on the palatal surface to accommodate the buccal force-delivery component. Thus, although attachment type and basic attachment geometry were kept constant, the spatial relationship between the attachment–aligner engagement site and the force-delivery component differed between the configurations. In addition, the buccal cut-out reduced local aligner coverage around the tooth and may have altered the contact and engagement geometry, potentially modifying force transmission. However, because attachment location, cut-out presence, and power-arm integration differed simultaneously, the observed difference should be interpreted as a configuration-dependent initial biomechanical response. The buccal vertical attachment configuration without a cut-out showed lower mesial rotation at each corresponding power-arm length compared with the palatal cut-out configuration.

Other recent finite element studies have examined different biomechanical variables related to attachment mechanics during molar mesialization. Pei et al. [13] investigated attachment design and tipping compensation, whereas Alsharif et al. [14] evaluated the effects of different attachment shapes and configurations on molar displacement and rotational behavior. Although these studies used different model designs and evaluated different outcome measures, they similarly demonstrate that modifications in attachment-related mechanics can influence the initial biomechanical response during clear aligner treatment.

Irrespective of attachment design, molar mesialization was consistently accompanied by mesial tipping and elevated periodontal ligament stress, indicating that attachment selection modulates but does not eliminate these effects. Within this shared pattern, the highest PDL stress values occurred in the scenarios with the shortest power arm configuration. These findings support the biomechanical concept that insufficient control over the force application point leads to unfavorable stress concentration within the periodontal ligament. In line with this biomechanical principle, Kang et al. [22] reported that short lever arms during extraction treatment with aligners resulted in higher PDL stress levels and undesirable tipping patterns. Collectively, these observations underscore the importance of simultaneously considering both tooth displacement patterns and periodontal ligament stress when designing force systems for molar mesialization in clear aligner therapy.

Across the tested power-arm lengths, a consistent length-dependent pattern was observed. In both attachment configurations, increasing power-arm length from 6 to 10 mm was associated with reductions in mesial tipping, peak PDL stress, and mesial crown displacement, whereas aligner deformation increased. Within the present two-step loading protocol, the increased aligner deformation should be interpreted as the combined mechanical response to the prescribed activation displacement and the elastic force generated by the TAD under the specific loading conditions of the model. However, the absolute differences were small, with ranges of 0.006 mm for mesial crown displacement, 0.015° for mesial tipping, and 0.025 MPa for peak PDL stress. These differences should therefore be interpreted primarily as biomechanical and directional findings rather than as clinically detectable treatment effects. The observed numerical differences should not be extrapolated directly to final clinical tooth positions. Rather, the consistent monotonic trend across both attachment configurations showed that increasing power-arm length was associated with reduced mesial tipping, although the clinical magnitude of this effect requires validation in longitudinal clinical studies.

Based on the directional trends observed in the present study, a preliminary biomechanical framework can be proposed for power-arm selection, pending clinical validation. A 6-mm power arm produced the greatest mesial crown displacement per activation and was associated with greater mesial tipping and higher PDL stress; it may be considered when greater mesial displacement per activation is prioritized, with careful monitoring of tooth angulation. An 8-mm power arm represents an intermediate option that may be considered when a balance between displacement and tipping control is desired, although the present data do not establish it as an optimal or universally preferable length. A 10-mm power arm produced the least mesial tipping and lowest PDL stress, but also the lowest mesial crown displacement per activation, and may therefore be considered when a lower initial tipping response is prioritized. These considerations are derived from single-activation biomechanical trends and are intended to guide clinical hypothesis generation rather than establish definitive treatment recommendations.

Several limitations should be considered when interpreting the present findings. First, the finite element model represents a deterministic simulation under predefined boundary and loading conditions and does not reproduce the time-dependent biological response associated with clinical tooth movement. The analysis was limited to a single aligner activation and consequently characterizes an initial biomechanical response rather than cumulative tooth movement over multiple aligners [23]. Second, the two attachment configurations differed simultaneously in attachment location, buccal cut-out presence, and power-arm integration. Therefore, the independent contribution of each component, including the potential effect of reduced aligner coverage associated with the cut-out, could not be isolated, and differences between configurations should be interpreted as configuration-dependent responses rather than effects attributable to any single component. Third, the TAD was maintained at a standardized position between the canine and first premolar at the level of the mucogingival junction to provide a reproducible point of force application while isolating power-arm length and attachment configuration as the primary experimental variables. Although this approach enabled direct comparison of the experimental configurations, individual anatomical variation in arch dimensions, inter-radicular space, and available insertion sites may require different TAD positions clinically. Changes in TAD position can alter the three-dimensional line of action of the applied force relative to the molar center of resistance and consequently modify the resulting moment-to-force relationship and tooth displacement pattern. Finally, the standardized material properties and geometric assumptions may not fully represent patient-specific anatomical and biological variability.

## Conclusions

Within the limitations of the present finite element model, increasing power-arm length from 6 to 10 mm was consistently associated with reduced mesial tipping and lower peak PDL stress, but also with reduced mesial crown displacement, representing a directional biomechanical trend rather than a clinically quantifiable effect. In all tested configurations, mesial crown displacement was accompanied by distal displacement of the mesiobuccal root apex, indicating a displacement pattern consistent with mesial tipping. The buccal vertical attachment configuration demonstrated lower mesial rotation than the palatal vertical attachment configuration with a buccal cut-out at each corresponding power-arm length; this difference should be interpreted as a configuration-dependent finding. These findings characterize the initial response to a single simulated aligner activation and should not be interpreted as evidence of long-term clinical tooth movement or treatment superiority.

## Data Availability

The datasets generated and analysed during the current study are included within the article. Additional data are available from the corresponding author on reasonable request.

## Abbreviations

CAT: Clear aligner therapy
CR: Center of resistance
FEA: Finite element analysis
PDL: Periodontal ligament
TAD: Temporary anchorage device
CT: Computed tomography
